# Oral rehabilitation of patients with cancer: challenges following surgical resection

**DOI:** 10.1007/s00520-026-11070-6

**Published:** 2026-08-19

**Authors:** Sérvulo da Costa Rodrigues Neto, Thaís Cristina Araújo Moreira, Marta Maria Alves Pereira, Mila Oliveira Santos Viana

**Affiliations:** 1 Federal University of Piauí, Campus Universitário Ministro Petrônio Portella, Bloco 5, Ininga, Teresina, Piauí 64049-550 Brazil; 2 University Hospital of the Federal University of Piauí, Teresina, Piauí Brazil

**Keywords:** Mouth neoplasms, Mouth rehabilitation, Surgery Oral

## Abstract

**Objective:**

To discuss the main challenges in oral rehabilitation of patients with oral cancer after surgical resection and to present viable strategies to overcome these limitations.

**Methods:**

A comprehensive literature search was conducted and the principal clinically relevant challenges identified were reduced bone height, flap volume, glossectomy, limited mouth opening, velopharyngeal dysfunction, and xerostomia.

**Results:**

Maxillary palatal ramp prostheses, mandibular guidance prostheses, and twin occlusion prostheses are indicated for patients without reconstruction grafting who present reduced bone height and mandibular deviation. In cases of extensive hard and soft tissue loss, type 3 fixed prostheses represent an appropriate option. Patients undergoing glossectomy may benefit from palatal or lingual augmentation and mandibular obturator prostheses. Sectional and flexible prostheses are suitable for individuals with restricted mouth opening. Maxillary or pharyngeal obturator prostheses are recommended for velopharyngeal dysfunction, while prostheses incorporating salivary reservoirs may assist patients with xerostomia.

**Conclusion:**

Oncologic treatment may result in multiple anatomical and functional oral alterations. Clinicians must be familiar with available prosthetic rehabilitation options to adequately manage these conditions. A multidisciplinary approach is essential to achieve effective and integrated care.

**Clinical significance:**

Presents practical, evidence-based alternatives to optimize function, comfort, and quality of life in patients with complex oral conditions.

## Introduction

Demographic projections estimate that the annual number of new cancer cases will reach 35 million by 2050 [1]. Oral cancer ranks among the most prevalent malignancies worldwide and continues to show an increasing incidence [2, 3]. It arises from structures within the oral cavity, including the gingiva, buccal mucosa, hard palate, floor of the mouth, salivary glands, and tongue [2, 3]. Major risk factors include tobacco use, alcohol consumption, and infection by human papillomavirus (HPV) [4]. Higher incidence rates are observed in men, older adults, and individuals of lower socioeconomic status [4].

The primary goal of oral cancer treatment is long-term disease control, typically achieved through surgery, with or without adjuvant radiotherapy or chemoradiotherapy [5]. Despite advances in treatment modalities, significant and often permanent changes in communication, appearance, nutrition, and oral function are common, resulting in substantial functional, psychological, and social impairments and decreased quality of life [2]. When functional or esthetic loss of bone structures occurs, reconstruction with free or microvascularized bone grafts is required to restore form and function, with dental rehabilitation representing the final stage of this reconstructive pathway [6, 7].

Although cancer management routinely involves a multidisciplinary team—including head and neck surgeons, oral and maxillofacial surgeons, and oncologists—the prosthetic rehabilitation phase predominantly depends on the dentist [8]. Prosthetic rehabilitation plays a fundamental role in reestablishing oral functions and helping patients overcome functional, esthetic, and social limitations [9]. Depending on the post-treatment oral condition, rehabilitation may involve removable or fixed prostheses, with the choice guided by the number of remaining teeth, the location and extent of bony and soft tissue defects, and overall oral anatomy following tumor therapy [10, 11].

Several factors may complicate prosthetic rehabilitation, such as inadequate prosthetic space, loss or scarring of soft tissues, flap volume and morphology, reduced bone height, hyposalivation, trismus, shallow or absent buccal vestibule, and loss of velopharyngeal competence [10, 12]. Additional anatomical changes resulting from treatment may include tooth loss, resection of structures such as the tongue or soft palate, alterations in muscle attachment and balance, and loss of labial competence [13]. Furthermore, temporomandibular joint dysfunction and changes in occlusal relationships may further impair masticatory rehabilitation [14].

Given the increasing global burden of oral cancer and its significant impact on oral functions, it is essential that dentists are adequately trained to address the complex rehabilitative needs of this population. Prosthetic rehabilitation is a key component of restoring oral functions and supporting patients’ reintegration into social life. However, this process is often hindered by challenges derived from both tumor characteristics and the consequences of oncologic treatment. Therefore, this study aims to discuss the primary challenges involved in the oral rehabilitation of patients with oral cancer who have undergone surgical resection and to present potential strategies to overcome them.

## Methods

A comprehensive literature search was conducted to identify, describe, and synthesize the available evidence on the main challenges associated with the oral rehabilitation of patients with oral cancer following surgical resection, as well as to explore potential strategies to address these challenges. This approach was chosen due to the broad and exploratory nature of the topic, which allows the integration of evidence from diverse study designs to provide a comprehensive overview of key aspects of rehabilitation in this population. Rather than addressing a narrowly defined clinical question, this review aimed to organize and critically discuss the available prosthetic options, thereby providing clinicians with a conceptual framework to support clinical decision-making. In addition, the existing literature is characterized by substantial heterogeneity in study designs, interventions, outcome measures, and reporting methods, which limits comparability across studies and precludes meaningful quantitative synthesis. In this context, a narrative approach was considered the most appropriate strategy to synthesize, contextualize, and critically interpret the available evidence, while enabling a comprehensive discussion of prosthetic options and their potential applications in the rehabilitation of patients with oral cancer.

### Search strategy

A comprehensive literature search was conducted in the PubMed and Scopus databases to identify studies addressing the prosthetic rehabilitation of patients treated for oral cancer. The search strategy combined controlled vocabulary and free-text terms related to oral cancer and prosthetic rehabilitation. The primary search string included (“oral cancer” OR “mouth neoplasms”) AND (“prosthetic rehabilitation” OR “prosthodontic rehabilitation” OR “oral rehabilitation”). No restrictions were applied with respect to publication year.

Following the initial screening of retrieved records, the most frequently reported clinical challenges affecting prosthetic rehabilitation were identified through an iterative analysis of the literature. These included reduced bone height and mandibular deviation, excessive flap volume, glossectomy-related defects, restricted mouth opening, velopharyngeal dysfunction, and xerostomia. Subsequently, additional targeted searches were performed by combining the primary search terms with challenge-specific descriptors, including “bone height,” “mandibular deviation,” “flap volume,” “glossectomy,” “restricted mouth opening,” “velopharyngeal dysfunction,” and “xerostomia.” The reference lists of all included studies and relevant review articles were also manually screened to identify additional publications not retrieved through the electronic search.

### Eligibility criteria

Studies were eligible for inclusion if they (1) included patients treated for oral cancer; (2) addressed prosthetic or prosthodontic rehabilitation following surgical and/or oncological treatment; and (3) reported clinical challenges, rehabilitation strategies, treatment outcomes, or considerations relevant to oral rehabilitation. Given the narrative nature of this review, original clinical studies, case series, case reports, narrative reviews, systematic reviews, and expert consensus papers were all considered eligible for inclusion. Eligibility was limited to articles published in English, with no restrictions on publication year.

Studies were excluded if they (1) focused exclusively on surgical reconstruction without addressing prosthetic rehabilitation; (2) involved head and neck cancers without providing specific data on oral cancer patients; or (3) were conference abstracts, editorials, letters to the editor, or non–peer-reviewed publications.

### Selection of sources of evidence

As this was a narrative review, study selection was performed by the authors based on scientific relevance, clinical applicability, and contribution to the understanding of challenges associated with prosthetic rehabilitation following oral cancer treatment. The selected studies were critically appraised, and relevant findings were synthesized and organized according to the major clinical challenges encountered in prosthetic rehabilitation. This narrative approach enabled the integration of diverse forms of evidence and provided a comprehensive overview of the factors influencing treatment planning and rehabilitation outcomes in this patient population.

## Clinical challenges after oral cancer treatment and prosthetic rehabilitation strategies

The clinically relevant challenges most frequently reported in the literature were identified, including compromised bone height and mandibular deviation [15–23], flap volume [10, 24–29], glossectomy [30–42], limited mouth opening [43–50], velopharyngeal dysfunction [30, 51–60], and xerostomia [61–68].

In patients undergoing bone resection without reconstruction using grafts, the residual bone height may be insufficient for the placement of osseointegrated implants [16], making rehabilitation with removable prostheses the indicated approach. However, mandibular deviation is another relevant complication that may occur in these cases, significantly compromising functional rehabilitation [15]. Reconstruction of oral cavity defects with soft tissue flaps is a common intervention following oncologic resections. These flaps may originate from different donor sites and, depending on their volume (whether insufficient or excessive), can interfere with prosthetic planning and rehabilitation [10, 25].

In cases of tumors involving the tongue and/or the floor of the mouth, partial or total glossectomy may be required, resulting in significant functional impairments, particularly in speech and swallowing [30]. Another relevant clinical challenge is limited mouth opening, resulting from alterations in the balance of the stomatognathic system after oncologic treatment, particularly in cases involving tissue loss and fibrosis formation. Furthermore, resection of tumors involving the soft or hard palate may create communication between the oral and nasal cavities, leading to velopharyngeal dysfunction [51]. Additionally, patients undergoing salivary gland removal or radiotherapy in the orofacial region may develop xerostomia, a condition that compromises the retention, stability, and comfort of dental prostheses [62].

**Fig. 1:**
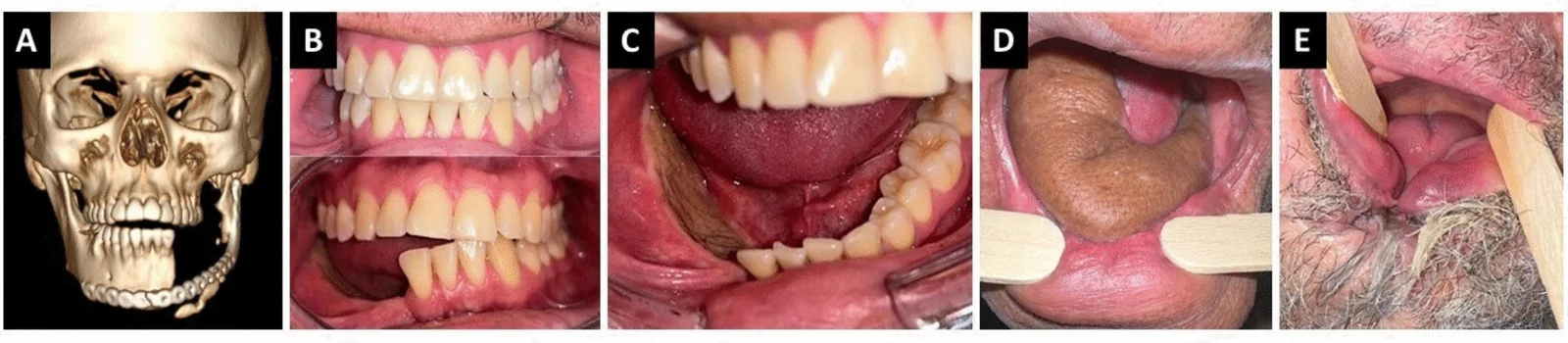
**A** Compromised bone height on the left side following mandibular resection. **B** The upper image shows stable occlusion prior to the surgical procedure; following resection, mandibular deviation is observed, as seen in the lower image. **C** The patient presented with combined soft- and hard-tissue loss following surgical resection and underwent reconstruction with a fibular free flap in the oral cavity to close the defect. **D** The patient underwent partial glossectomy, and a bulky flap used for reconstruction of the floor of the mouth is still observed. **E** The patient presents with limited mouth opening following tissue loss resulting from oncologic treatment. Images provided by the authors

Figure 1 illustrates some of the main challenges described above. The prosthetic rehabilitation strategies applicable to these situations will be discussed below.

### Compromised bone height and mandibular deviation

Following segmental mandibulectomy without reconstruction of hard tissue, masticatory function becomes impaired due to mandibular deviation toward the resected side (Fig. 1B) [15]. Although surgical reconstruction with grafts and implants is the ideal approach, this is not feasible for all patients (Fig. 1A); therefore, alternative prosthetic strategies are required to restore esthetics and function through the use of removable prostheses [16].

**Fig. 2:**
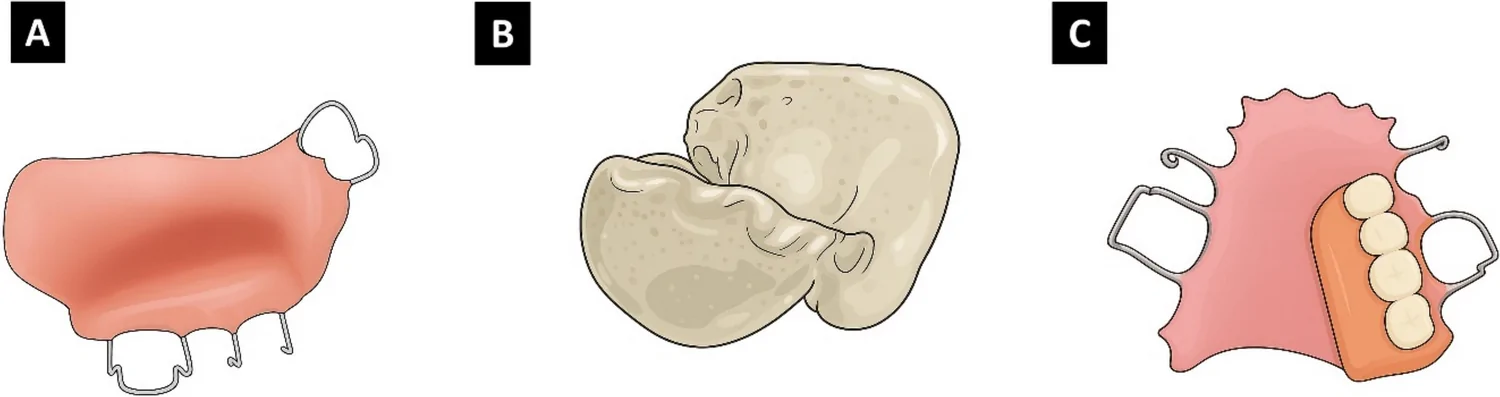
**A** Maxillary palatal ramp prosthesis. **B** Mandibular guidance prosthesis. **C** Twin occlusion prosthesis. The schematic representations of the prostheses were adapted from Sharma et al*.* (2022)^18^. Created in BioRender. Alves Pereira, M. M. (2026) https://BioRender.com/b6upx1q

In such cases, a maxillary palatal ramp prosthesis (Fig. 2A) can be fabricated to maintain a functional maxillomandibular position, improving speech and swallowing by guiding the mandibular teeth into an intercuspal position during closure. Palatal ramps or guide planes made from autopolymerizing acrylic resin may be added to the maxillary prosthesis to assist in mandibular positioning [17]. Similarly, a mandibular guidance prosthesis (Fig. 2B) can limit deviation toward the defect side by contacting the buccal surfaces of the maxillary posterior teeth, promoting proper overlap and functional closure [18]. In addition to stabilizing the prosthesis and limiting mandibular deviation, the maxillary ramp provides a broad occlusal platform that enhances masticatory efficiency [15]. To prevent interference with speech, swallowing, and other oral functions, the ramp should not extend below the occlusal level of the maxillary teeth [18]. Moreover, an acrylic guide flange prosthesis represents a simple, conservative, and cost-effective approach for managing mandibular deviation [17].

When manual repositioning of the mandible allows the achievement of an acceptable occlusal relationship, guidance prostheses may be used as an interim therapeutic strategy until a stable occlusion is established and definitive rehabilitation with a removable partial denture becomes feasible [15, 17, 18]. During this period, early prosthetic intervention aims to minimize mandibular deviation, prevent extrusion of the maxillary dentition, and improve masticatory efficiency [19]. Bhattacharya et al. [17] reported successful correction of mandibular positioning within 3 weeks using a palatal ramp prosthesis, whereas Marathe and Kshirsagar [16] observed marked improvement in mandibular deviation after 4 weeks of treatment with a mandibular guidance prosthesis.

Nevertheless, mandibular guidance therapy demonstrates greater effectiveness in patients whose surgical resection is confined to osseous structures with minimal involvement of the surrounding soft tissues [17, 19]. Conversely, delayed prosthetic intervention, particularly in the presence of extensive scar formation and soft tissue contracture, may compromise or even preclude the fabrication and effectiveness of a guidance prosthesis [20].

An alternative rehabilitation strategy is the twin occlusion prosthesis (Fig. 2C), which incorporates two rows of artificial teeth: one positioned conventionally within the edentulous ridge and a second located on the palatal aspect, thereby creating an enlarged functional occlusal surface [21, 22]. This design is particularly advantageous for patients with limited financial resources who lack access to reconstructive surgery or implant-supported rehabilitation [22].

For patients with mandibular deviation in whom manual achievement of an acceptable occlusion is not possible, a twin occlusion prosthesis is the preferred treatment option [15]. In these cases, severe scar contracture and fibrosis of the residual tissues often prevent the establishment of a stable interocclusal relationship, even with the use of guidance prostheses. Under such circumstances, the maxillary twin occlusion prosthesis offers an effective alternative for improving mastication and esthetics, although it does not completely eliminate mandibular deviation [15, 18]. This prosthetic design is also indicated for patients with insufficient abutment teeth or for completely edentulous individuals in whom guidance prostheses cannot be retained, as the additional occlusal table contributes to improved prosthesis stability [16, 23]. Clinical reports by Marathe and Kshirsagar [16] and Agarwal et al. [20] demonstrated favorable functional outcomes, with patients reporting improved masticatory efficiency and high levels of treatment satisfaction. These improvements were maintained throughout follow-up periods of 4 years and 6 months, respectively, during which proper intercuspation of the mandibular teeth was successfully achieved.

### Flap volume

Although surgeons strive to minimize anatomical deformities during tumor resection, minor pre-prosthetic soft tissue corrections may be necessary in some cases to eliminate unacceptable interference caused by residual scar tissue [24]. Flaccidity or excess bulk of a thick flap may restrict prosthetic space or compromise retention (Fig. 1D), requiring secondary procedures such as flap volume reduction or suspension to improve support [25]. The use of anchoring devices allows local flaps to be fixed directly to the alveolar ridge bone, ensuring proper positioning and firm attachment both of which are prerequisites for achieving a functional outcome after cancer reconstruction [26].

Flap suspension using anchoring devices offers several clinical advantages, including technical simplicity, fewer anatomical restrictions during positioning, and easier intraoperative adjustment of suture tension. These characteristics facilitate secure soft tissue stabilization while providing sufficient mechanical strength for reliable ligament fixation [25]. In the study by Abe et al. [25], no recurrence of flap ptosis or wound infection was observed after 3 years of follow-up. Furthermore, the patient reported high treatment satisfaction, with restoration of chewing ability, effective food bolus formation, and normal swallowing without prosthesis instability or tooth dislodgement.

In other cases, surgical intervention is required to reestablish anatomy that has healed in a configuration incompatible with prosthesis adaptation. These procedures help eliminate fibrous scar tissue resulting from cancer resection, enhance muscle mobility, and deepen the sublingual sulcus and vestibular fornix, thereby facilitating prosthesis placement and stability [27].

Romeo et al. [24] reported the rehabilitation of a patient presenting with fibrous scar tissue extending between the tongue and the alveolar ridge mucosa, which severely restricted tongue mobility and impaired speech articulation. Surgical soft tissue correction was performed, resulting in complete closure of the surgical defect within 28 days and enabling subsequent prosthetic rehabilitation. No postoperative discomfort or recurrent scar tissue formation was observed. Following an initial adaptation period, the patient reported improved masticatory function after adopting the new dietary regimen and expressed a high degree of satisfaction with the treatment.

Before considering implant placement or surgical soft tissue correction, several additional factors should be carefully evaluated, including the radiation dose, implant location, extent of the irradiated field, proximity to critical anatomical structures, and the patient’s systemic health status [10, 24].

**Fig. 3:**
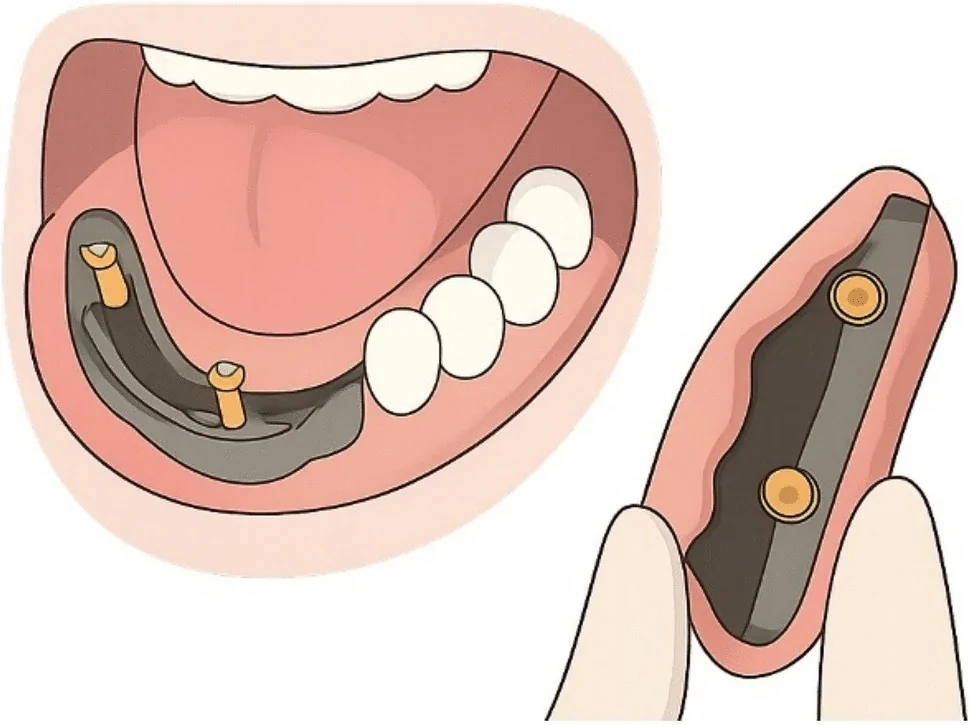
A type III fixed prosthesis is indicated in cases involving combined soft- and hard-tissue loss, adapted from Manju et al*.* [10]. Created in BioRender. Alves Pereira, M. M. (2026) https://BioRender.com/g5zqjf1

Conversely, in cases involving extensive loss of hard and soft tissue (Fig. 1C), a type 3 fixed prosthesis is an ideal option (Fig. 3), as it replaces both dental crowns and lost soft tissue using pink restorative materials [10]. When the interarch space is increased, conventional implant-supported porcelain-fused-to-metal prostheses become less feasible due to the risk of metal distortion in large frameworks [27]. In such situations, a hybrid prosthesis, composed of a metal substructure with an acrylic overlay, is recommended. This design reduces stress concentration within the system, improves masticatory performance, and decreases the risk of fracture at the bone–implant interface under heavy occlusal load [10].

Hybrid prostheses are considered one of the most cost-effective fixed rehabilitation options because they are associated with relatively simple fabrication procedures, a low incidence of technical complications, favorable esthetic outcomes, and greater patient satisfaction and masticatory performance than conventional implant-supported overdentures [10, 27]. In addition to effectively replacing missing soft tissues, the shock-absorbing properties of acrylic resin may help minimize both mechanical and biological complications, including prosthetic component fracture, screw loosening, and peri-implant bone resorption [28].

Despite these advantages, hybrid prostheses also present important limitations. Their considerable bulk requires substantial restorative space, generally at least 15 mm, while the prosthetic contours may not accurately reproduce the morphology of the natural dentition [10, 27]. In addition, the increased prosthetic volume may adversely affect speech, favor food accumulation beneath the prosthesis, and predispose the acrylic teeth to staining over time [10, 27]. It should also be emphasized that hybrid prostheses are indicated as fixed implant-supported restorations only in patients reconstructed with bone grafts. Furthermore, all FP-3 prostheses exhibit increased crown height, making it essential to maintain sufficient clearance between the prosthesis and the underlying soft tissue to permit effective use of interdental hygiene devices and facilitate long-term maintenance [10].

Akifuddin and Awadalkreem [29] reported the successful rehabilitation of a patient presenting with excessive interarch space following resection of an oral squamous cell carcinoma using a hybrid implant-supported prosthesis. After 5 years of follow-up, all implants remained functional, resulting in a 100% implant survival rate without prosthetic fracture or implant loss. Excellent peri-implant soft tissue health and outstanding prosthetic stability were maintained throughout the observation period, while the patient reported substantial improvements in oral function, comfort, and overall treatment satisfaction.

### Glossectomy and dysphagia

The functional ability to swallow, articulate speech, and chew is directly influenced by the extent of tongue resection (Fig. 1D), since the tongue must contact the palate to perform these functions [30]. Patients undergoing partial glossectomy may benefit from palatal prostheses designed to reduce the palatal contour, while total glossectomy usually requires a mandibular lingual prosthesis to reduce the size of the oral cavity, thereby improving speech and directing food toward the esophagus to facilitate swallowing [31].

**Fig. 4:**
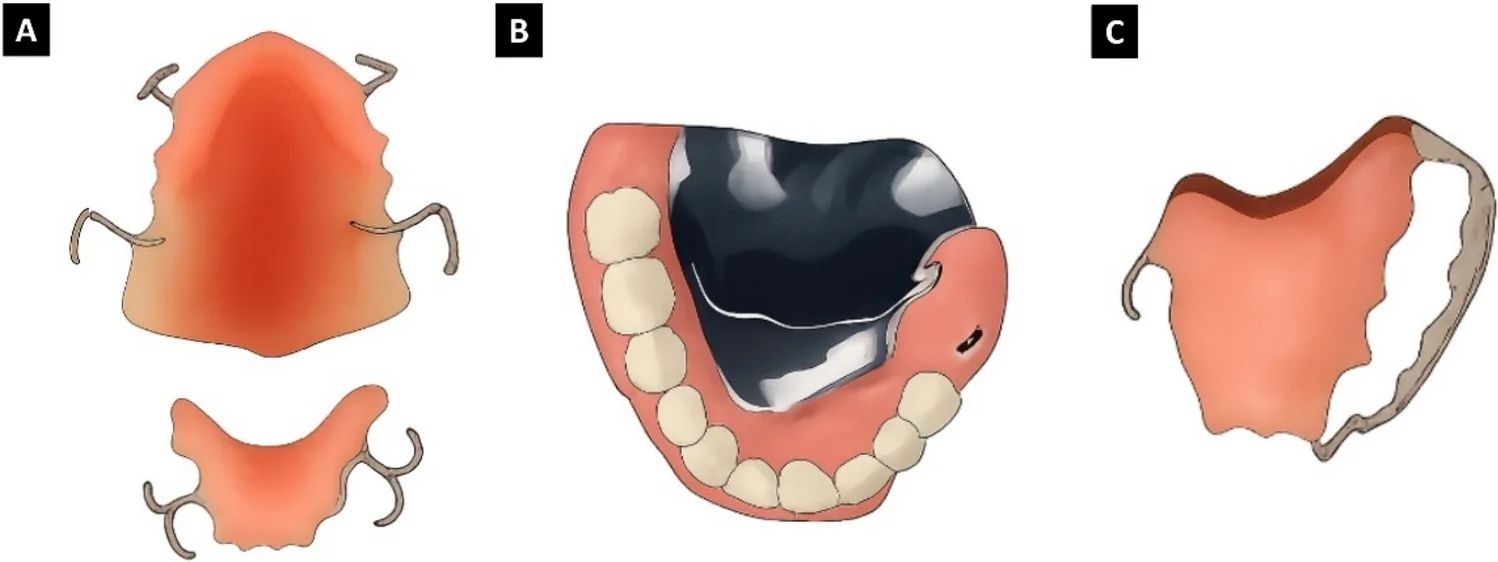
**A** Palatal and lingual augmentation prosthesis, adapted from Ohno and Fujishima [33]. **B** Mandibular prosthesis with a lingual plate, adapted from Penn et al*.* [40]. **C** Mandibular obturator prostheses, adapted from Jagtiani et al*.* [41]. Created in BioRender. Alves Pereira, M. M. (2026) https://BioRender.com/9s82a8l

When tongue elevation and palatal contact are compromised, a palatal augmentation prosthesis (PAP) can lower the palatal vault to meet the residual tongue, improving speech and swallowing by increasing contact surface area [30]. The PAP (Fig. 4A) can also be used in the management of dysphagia, as it reshapes the hard palate to increase tongue–palate contact during swallowing, improving tongue pressure and reducing residual food in the oral and pharyngeal cavities [32]. A lingual augmentation prosthesis (Fig. 4A) can also aid in tongue movement and in the correction of dysphagia [33].

Patients who undergo partial glossectomy involving less than 50% of the tongue generally experience minimal functional impairment and therefore rarely require prosthetic rehabilitation. In contrast, resection exceeding 50% of the tongue usually necessitates rehabilitation with either a palatal augmentation prosthesis or a lingual prosthesis. The palatal augmentation prosthesis, also referred to as a palatal drop prosthesis, is particularly indicated when tongue resection and reconstruction result in reduced tongue bulk and restricted mobility of the reconstructed tissue [34].

Evidence from two systematic reviews demonstrates that treatment with a PAP significantly alleviates impairments in speech and swallowing while improving patients’ quality of life [35, 36]. Nevertheless, although many patients perceive speech production to require less effort following rehabilitation, these improvements are not always recognized as clinically significant by external listeners [37]. Furthermore, some individuals experience discomfort or discontinue use of the prosthesis because of altered taste perception and other sensory disturbances [38].

Bachher and Dholam [39] described the successful use of a lingual prosthesis over a 12-year period in a patient who had undergone total glossectomy, reporting improved swallowing through more efficient food bolus control. However, excessive salivation remained a challenge and required adjunctive oral exercises for management. Despite these favorable outcomes, the functional performance of lingual prostheses remains inherently limited because their rigid structure lacks the mobility necessary to reproduce the highly coordinated and dynamic movements of the native tongue [39, 40].

When the tongue is completely resected along with the floor-of-mouth muscles, only the esophageal phase of swallowing remains intact, and thus the functional benefit of a palatal augmentation prosthesis is limited. In such cases, a mandibular prosthesis with a lingual plate (Fig. 4B) may be designed, featuring a funnel-shaped base that guides food toward the esophagus and supports the collapsed lower lip [40].

Penn et al. [40] reported the successful use of this prosthesis in a patient following total glossectomy. After 2 years of follow-up, the patient demonstrated improved mastication and regained the ability to ingest solid foods, eliminating the need for nasogastric tube feeding. In addition, the feeding-aid prosthesis maintained the occlusal vertical dimension, thereby improving facial support and overall facial appearance. Its principal limitation, however, lies in the greater complexity of fabrication, which requires more extensive laboratory procedures and consequently increases treatment costs.

Additionally, a mandibular obturator prosthesis (Fig. 4C) made of acrylic resin may also be beneficial to reduce salivary incontinence, improve swallowing and articulation, and aid in the transition to oral feeding [41]. This approach is particularly valuable when secondary flap surgery to repair the fistula carries its own associated risks, including infection, donor-site morbidity, flap failure, and the formation of a new fistula or recurrence of the original fistula [42]. Jagtiani et al. [41] reported a case series involving three patients followed for 1 year in whom mandibular acrylic obturator prostheses were well tolerated, effectively eliminated dead space, avoided the morbidity associated with additional surgical procedures, reduced salivary incontinence, improved swallowing and speech articulation, and enabled the successful resumption of oral feeding. Given the complexity of these prostheses, comprehensive instructions regarding their use, maintenance, and hygiene should always be provided to both patients and caregivers.

Importantly, patients treated for oral cancer remain frequently underserved with respect to rehabilitation of treatment-related sequelae, including speech impairment, sialorrhea, and feeding difficulties. Many are never evaluated by, or referred to, a speech-language pathologist despite the substantial functional deficits associated with glossectomy [37]. Therefore, speech-language therapy and orofacial myofunctional rehabilitation should be integrated into prosthetic treatment protocols, as prosthetic rehabilitation alone is often insufficient to fully restore articulation, speech intelligibility, and prosodic function [36].

### Restricted mouth opening

Prosthetic rehabilitation of patients with restricted mouth opening (Fig. 1E), a possible consequence of oncologic treatment, complicates all stages of prosthetic rehabilitation, from preliminary impressions to prosthesis insertion [43].

The reduced ability of patients with limited mouth opening to maintain adequate oral hygiene may place them at an increased risk of infection, particularly when wearing a prosthesis that requires additional maintenance. Simplifying the prosthesis design and implementing interventional oral hygiene maintenance programs may contribute to the longevity and success of any prosthesis intended for the rehabilitation of these patients [44]. In cases where microstomia cannot be managed by surgery or dynamic mouth-opening devices, modified impression procedures and prosthesis design facilitate prosthetic rehabilitation [43].

**Fig. 5:**
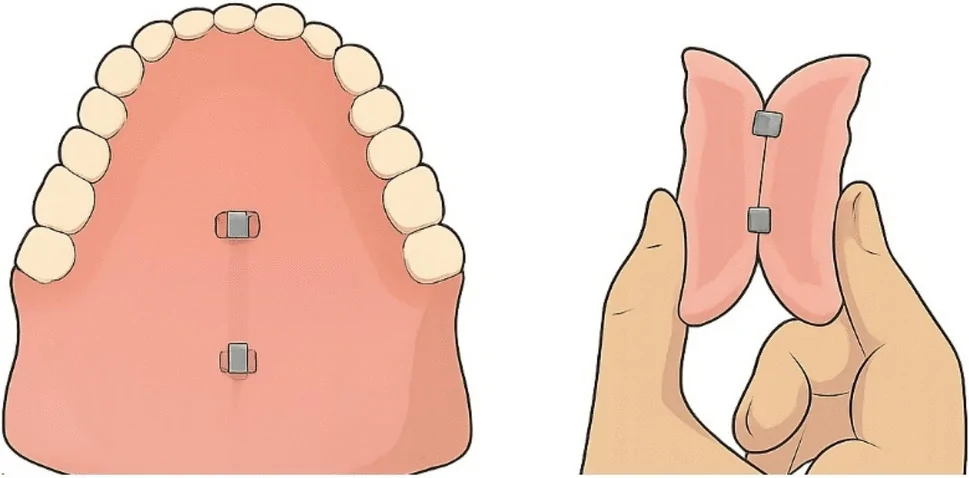
Sectional prosthesis, adapted Sharma et al*.* [43]. Created in BioRender. Alves Pereira, M. M. (2026) https://BioRender.com/eeobdht

The literature describes several modified impression techniques using flexible or sectional trays, as well as adapted prosthetic designs, including sectional, flexible, collapsible, and articulating prostheses with various locking or attachment mechanisms developed to overcome this limitation [44]. Sectional prostheses (Fig. 5) provide ease of insertion and removal, cost-effectiveness, and maximum coverage for support, retention, and stability. However, the increased laboratory work required is a notable limitation of this technique [45].

Celakil et al. [46] reported the successful use of a sectional mandibular prosthesis in a patient with radiation-induced trismus. The patient expressed satisfaction with the insertion technique, and clinical follow-up for more than 1 year demonstrated favorable functional outcomes. Nevertheless, this approach presents several limitations, including reduced tongue space and increased laboratory workload resulting from the incorporation of additional components, such as hinges and specialized attachment systems, during prosthesis fabrication [47, 48]. Moreover, successful long-term use depends heavily on patient cooperation, as routine insertion and removal require adequate manual dexterity and psychomotor coordination [47, 48].

In most cases, custom sectional trays are fabricated in two parts using autopolymerizing acrylic resin and joined along the midline using locking mechanisms, ranging from swing-lock systems to simple hinge attachments. Flexible prostheses, on the other hand, are made of resilient materials such as Valplast, allowing for easy insertion and removal [44].

Despite these advantages, the use of flexible materials requires careful patient selection and sound clinical judgment because concerns remain regarding stress distribution, color stability, and long-term hygiene maintenance [49]. Nevertheless, flexible prostheses may represent an effective rehabilitation option for socioeconomically disadvantaged patients in whom advanced treatments, such as implant-supported rehabilitation or bone grafting procedures, are not feasible, providing functional and esthetic improvements while facilitating prosthesis insertion and removal [50]. In addition, because sectional prostheses require more complex and time-consuming laboratory procedures, flexible prostheses may be preferable when rapid rehabilitation is required [49].

Egan and Swindells [49] described the rehabilitation of a patient who had undergone three surgical procedures involving the lower lip for squamous cell carcinoma and presented with a maximum mouth opening of only 30 mm. Owing to the severe microstomia, the patient’s conventional mandibular prosthesis could no longer be inserted, resulting in considerable functional impairment. During a 6-month follow-up period, the flexible prosthesis demonstrated satisfactory retention, esthetics, and patient comfort, while allowing straightforward insertion for both the patient and the clinician.

A systematic review evaluating prosthetic rehabilitation in patients with microstomia of various etiologies, including surgical complications, reported a mean maximum mouth opening of 22.7 mm. The most frequently employed rehabilitation approaches were sectional, collapsible, flexible, and foldable prostheses (41.1%), followed by implant-supported prostheses (14.7%). The mean follow-up period across the included studies was 17.54 months. Most patients adapted successfully to their prostheses without major complications. Based on the available evidence, the authors concluded that any of these prosthetic approaches may be successfully employed for the rehabilitation of patients with microstomia, provided that treatment selection is individualized according to the patient's functional requirements and the severity of the restricted mouth opening [44].

### Velopharyngeal dysfunction

Velopharyngeal dysfunction may result from soft palate defects, including those acquired after tumor resection [51]. Loss of velopharyngeal integrity can lead to speech problems, particularly hypernasal resonance caused by excessive nasal air escape, as well as swallowing difficulties [30].

**Fig. 6:**
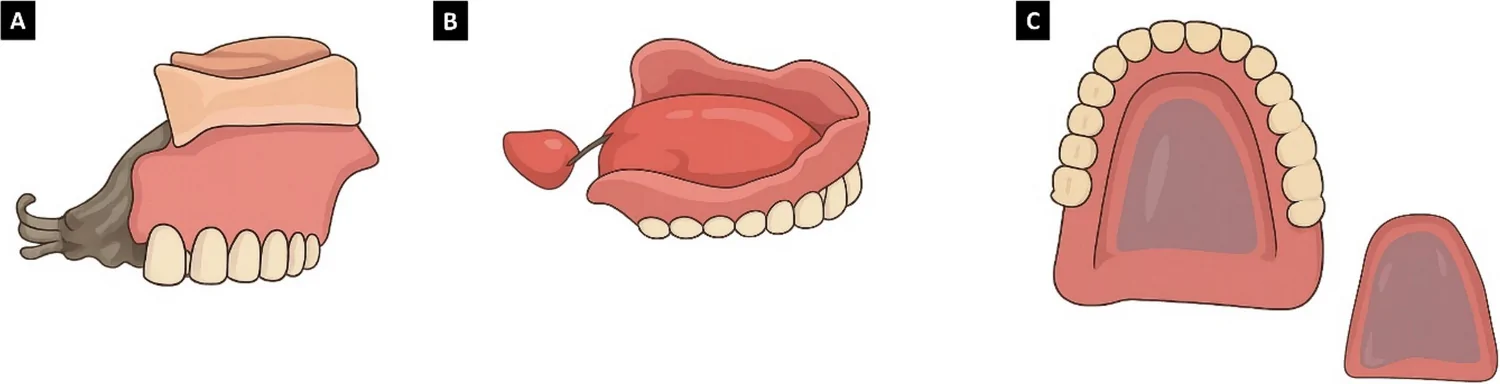
**A** Maxillary obturator prostheses, adapted from Nayar [30]. **B** Pharyngeal obturator, adapted from Naveen et al*.* [53]. **C** Prostheses incorporating salivary reservoirs, adapted from Srivastava et al*.* [63]. Created in BioRender. Alves Pereira, M. M. (2026) https://BioRender.com/9ko47to

Prosthetic treatment is achieved through the use of an obturator prosthesis (Fig. 6A), a removable maxillary device with a posterior extension designed to separate the oropharynx from the nasopharynx, thereby correcting soft palate defects and enabling proper closure of the palatopharyngeal sphincter [51]. The use of an obturator prosthesis improves swallowing by reestablishing a more natural pattern of liquid ingestion and enhances speech intelligibility by reducing nasal resonance [52]. Patients with minor soft palate defects may benefit from a conventional complete denture featuring a posterior pharyngeal bulb extension (Fig. 6B) [53].

It is important to emphasize that the primary function of the obturator prosthesis is to control air emission during speech and prevent the leakage of food or liquids into the nasal cavity during swallowing. However, speech improvement is only possible when the prosthesis is used in conjunction with speech therapy provided by a speech-language pathologist [54]. Obturator prostheses are particularly indicated for individuals with oral or systemic conditions that contraindicate surgical repair [53].

Among the principal advantages of obturator prostheses is their ability to eliminate the need for a second surgical procedure for defect closure while providing immediate restoration of oral function and facial morphology. An additional benefit is the ease of clinical inspection of the surgical site, facilitating early detection of local tumor recurrence during routine follow-up examinations [55]. Furthermore, because some patients are not suitable candidates for surgical reconstruction owing to oral or systemic contraindications, obturator prostheses often represent the most appropriate therapeutic option for restoring mastication, swallowing, and speech [53].

Achieving satisfactory retention with conventional prostheses remains particularly challenging in edentulous patients presenting with combined hard and soft palate defects because prosthesis weight and the inability to establish an adequate peripheral seal compromise stability [53]. The posterior extension of the obturator creates a long lever arm, increasing prosthesis weight, gravitational forces, rotational movement around the fulcrum, and the mechanical demands placed on the remaining supporting structures [56]. Consequently, many patients fail to recover preoperative levels of speech and swallowing function despite prosthetic rehabilitation [52]. Patient discomfort also tends to increase when both the hard and soft palate have been resected [51]. Careful attention should likewise be paid to prosthesis positioning. Placement superior to the level of pharyngeal wall movement may obstruct the nasopharynx, resulting in nasal airway obstruction and hyponasal speech, whereas excessive inferior positioning within the oropharynx may interfere with tongue function and induce gagging [56].

Despite their well-established clinical benefits, obturator prostheses present important limitations, including inadequate sealing with incomplete oronasal separation and reduced stability resulting from the lack of sufficient supporting tissues. These challenges become progressively greater in patients with extensive posterior defects or a completely edentulous residual maxilla [57].

Tuna et al. [51] reported the successful rehabilitation of a patient following resection of a soft palate squamous cell carcinoma using an obturator prosthesis. Adequate velopharyngeal closure was achieved without evidence of nasal regurgitation. Assessment by a speech-language pathologist demonstrated reduced hypernasality based on oral and nasal consonant production, while perceptual speech evaluation revealed marked improvement in speech intelligibility. After 3 years of follow-up, the patient remained highly satisfied with the prosthesis [51]. Nevertheless, meaningful improvements in speech can only be achieved when prosthetic rehabilitation is integrated with speech therapy provided by a speech-language pathologist [54].

Systematic reviews comparing surgical reconstruction with prosthetic obturation following maxillectomy have demonstrated comparable improvements in quality of life and overall health-related outcomes between both treatment approaches [58, 59]. Nevertheless, surgical reconstruction may offer specific advantages in selected clinical situations [59]. Therefore, treatment selection should be individualized according to multiple factors, including defect location and extent, preservation of strategically important teeth, and the anticipated risk of tumor recurrence [58].

Although obturator prostheses have traditionally been recommended for small defects, growing evidence indicates that they may provide functional outcomes comparable to those achieved with surgical flap reconstruction, even in patients with more extensive defects [59]. However, larger defects are frequently associated with fewer remaining teeth, diminished supporting tissues, and greater prosthetic demands, making rehabilitation more complex. In these situations, the increased vertical extension required to close the defect results in a heavier prosthesis, reduced patient comfort, and greater difficulty during functional adaptation. When sufficient bone quantity and quality are available, implant-retained obturator prostheses may provide enhanced retention, stability, and overall prosthetic performance, thereby minimizing many of these biomechanical limitations [60].

### Xerostomia

Radiation-induced xerostomia is a significant complication in patients with head and neck cancer, as radiation causes irreversible damage to the salivary glands within the exposed region [61]. Dentures are often poorly tolerated by these patients because saliva acts as a thin lubricating film between the prosthesis and the oral mucosa; its absence can result in reduced retention and an increased risk of inflammation and ulceration in the oral cavity [62]. One of the therapeutic approaches involves the use of saliva substitutes, which improve lubrication and act as moisturizing agents for the dry mucosa, thereby enhancing the retention and comfort of dental prostheses [63]. Nevertheless, maintaining the continuous delivery of salivary substitutes without interfering with patients’ daily activities remains one of the greatest challenges associated with this approach [62].

Complete dentures containing a palatal reservoir filled with an artificial saliva (Fig. 6C) can be fabricated to improve oral health and quality of life in edentulous patients suffering from xerostomia, while helping to prolong oral moisture [61]. Continuous administration of saliva substitutes can be achieved over an extended period using saliva reservoir dentures, which do not require complex laboratory procedures or sensitive fabrication techniques [63].

The amount of available interarch space is the primary factor that should be considered before fabricating a maxillary reservoir prosthesis. In patients presenting with a shallow palatal vault, incorporation of a palatal reservoir is generally contraindicated. The principal limitation of this design is the increased palatal thickness, which reduces the functional oral cavity volume and may negatively affect swallowing and speech [63]. Conversely, maxillary reservoir prostheses offer several important advantages, including direct visualization of the saliva substitute within the reservoir chamber and easy access for refilling during clinical appointments [64]. Furthermore, these prostheses can be fabricated using conventional denture materials, are relatively easy to maintain hygienically, and provide effective lubrication of the oral tissues [63]. Compared with mandibular reservoirs, maxillary reservoirs also provide greater storage capacity and allow the saliva substitute to distribute more uniformly throughout the oral cavity, thereby reducing the likelihood of outlet obstruction by food debris or oral fluids. In contrast, mandibular reservoirs primarily direct the lubricant toward the floor of the mouth [65].

For mandibular rehabilitation, a two-piece complete denture with a built-in reservoir may be fabricated, consisting of an upper part (containing the artificial teeth) and a lower part (containing the saliva reservoir) connected by retentive attachments and featuring lingual drainage openings for the controlled release of the saliva substitute [62]. Notably, the continuous flow of saliva from the reservoir enhances patient comfort, provides adequate lubrication of food to facilitate swallowing, helps maintain oral pH balance, and contributes to greater prosthesis retention [63].

An important advantage of sectional reservoir prostheses is the ease of access to the reservoir chamber for cleaning and refilling. The use of a transparent acrylic denture base further facilitates determination of the optimal reservoir size and position during fabrication while allowing both the clinician and the patient to monitor the remaining volume of saliva substitute [66]. However, these prostheses also present important limitations. Adequate vertical restorative space is required to accommodate the reservoir, which may compromise the mechanical strength of the prosthesis. In addition, repair and relining procedures become considerably more challenging [67]. Successful long-term use also depends on sufficient patient manual dexterity to separate and reassemble the prosthetic components. Furthermore, fabrication is technically demanding, requiring meticulous laboratory procedures, while inadequate maintenance may promote fungal colonization and obstruction of the outlet openings by accumulated food debris or oral fluids [66].

Joseph et al. [65] reported the successful fabrication of a maxillary complete denture incorporating a salivary reservoir for a patient with radiation-induced xerostomia. After 6 months of follow-up, the patient reported marked relief of xerostomia symptoms and found the prosthesis easy to use and maintain. Similarly, Ladda et al. [68] described the fabrication of a mandibular reservoir prosthesis for a patient with radiotherapy-induced xerostomia following treatment for oral carcinoma. The patient successfully wore the prosthesis with satisfactory retention for more than 2 years, reporting sustained relief of oral dryness together with improved food intake.

In patients presenting with xerostomia and limited vertical restorative space, flexible prostheses may represent an alternative rehabilitation strategy. Compared with conventional acrylic dentures, these prostheses are lighter, more resistant to deformation and fracture, and can be fabricated with minimal thickness. In addition, their resilient material adapts more effectively to the continuous movement and flexibility of the oral tissues while maintaining adequate stability during mastication, including the chewing of harder foods. Their principal limitation, however, is the higher cost of flexible resin materials compared with conventional acrylic resins [67].

**Fig. 7:**
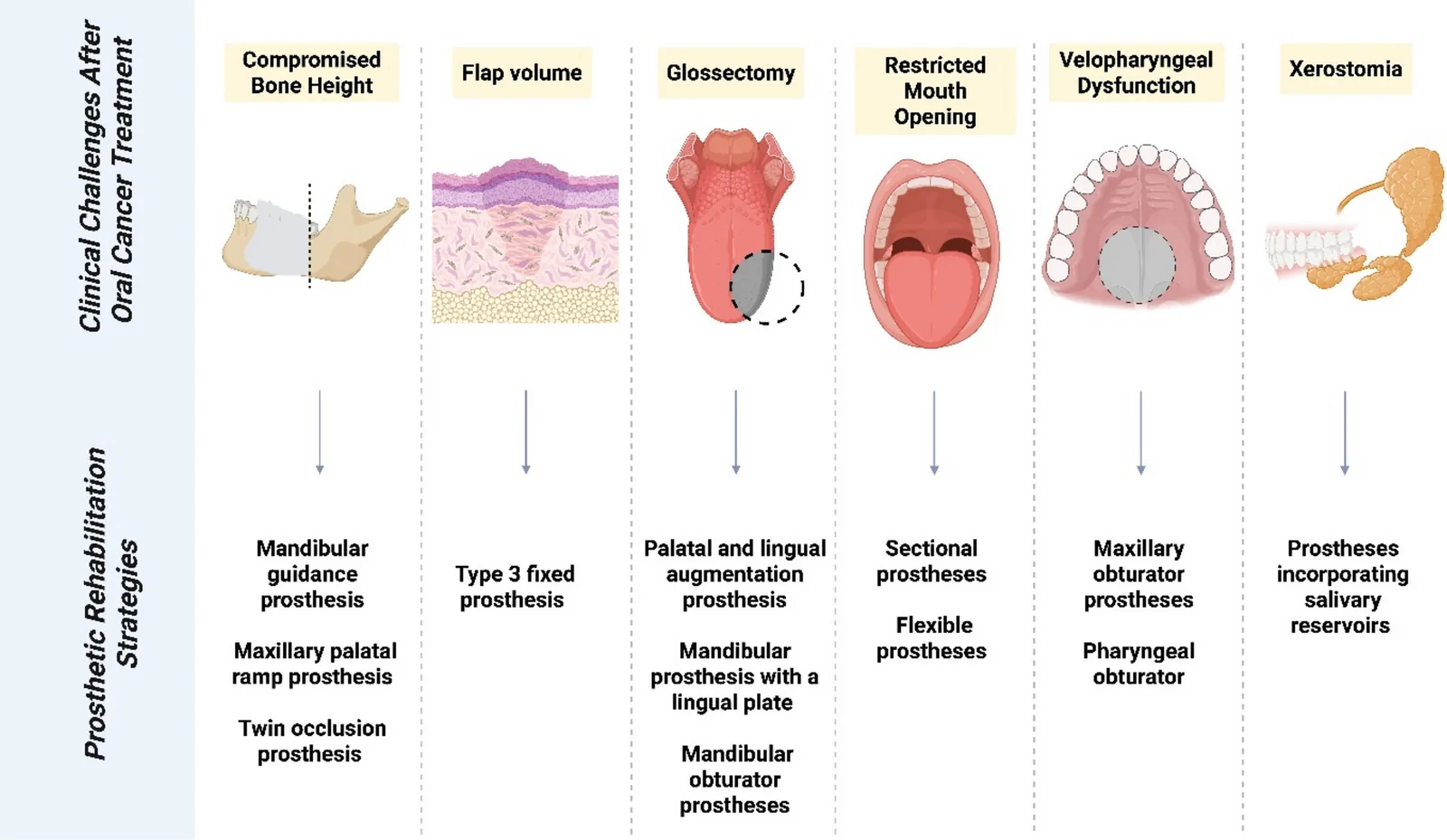
Schematic representation of the main challenges involved in the prosthetic rehabilitation of patients treated for oral cancer, as well as the strategies described in the literature to overcome these limitations and achieve functional and esthetic rehabilitation. Created in BioRender. Alves Pereira, M. M. (2026) https://BioRender.com/l5ojx2y

The principal challenges encountered during oral rehabilitation following oncologic treatment for oral cancer, together with the corresponding prosthetic strategies discussed throughout this review, are summarized in Fig. 7.

### Advances and future perspectives

Prosthetic rehabilitation of ablative defects remains a significant clinical challenge because of the unique anatomical, functional, and esthetic complexities encountered in maxillofacial patients [69]. With advances in digital technologies, the methods used for the fabrication of intraoral maxillofacial prostheses continue to evolve and improve. Digital technologies provide complementary support or, in some cases, integrate all stages of the fabrication process for intraoral maxillofacial prostheses [70]. CAD/CAM techniques offer several advantages, including superior dimensional accuracy, greater reproducibility through automated manufacturing processes, reduced patient discomfort by eliminating conventional impression materials, significantly shorter turnaround times from data acquisition to prosthesis delivery, and reduced need for post-delivery adjustments [69].

Fully digital workflows for maxillary and mandibular intraoral prostheses are still in the early stages of development, with hybrid workflows remaining the most commonly adopted approach [69]. Hybrid workflows are particularly advantageous because they combine the precision of CAD software with the versatility of three-dimensional (3D) printing technology, enabling clinicians to produce highly accurate patient-specific positive casts that serve as the basis for subsequent conventional prosthesis fabrication [70].

Trismus, xerostomia, and mucositis frequently complicate conventional prosthetic procedures. However, digital impression techniques can effectively overcome many of these challenges [71]. Digital impressions obtained with intraoral scanners can significantly reduce laboratory working time, minimize the risk of aspiration of impression materials, and provide greater patient comfort [72].

When feasible, osseointegrated implants represent the most advanced treatment modality for craniofacial reconstruction [73]. Nevertheless, implant rehabilitation may be compromised by insufficient bone volume, poor soft tissue quality, and the presence of delicate vascularized free flaps, making implant-supported rehabilitation technically demanding and often unpredictable [73]. Digital transformation has introduced guided implant surgery through the use of prefabricated surgical guides that ensure accurate implant positioning [74]. Implant placement, which represents the final stage of oncologic reconstruction, can therefore be precisely planned and executed using a digital workflow [75]. Such accuracy is clinically relevant in patients with oral cancer, in whom the available bone segments are often limited and implants must be placed within millimeters of reconstructive plates or vascular pedicles [74].

Bone tissue is particularly susceptible to the effects of radiotherapy, resulting in hypocellular, hypovascular, and hypoxic bone, which leads to delayed and impaired healing and increases the risk of osteoradionecrosis [76]. Nevertheless, according to published meta-analyses, implants placed in irradiated bone demonstrate clinically satisfactory survival rates after 5 years (93.13%), although these rates remain lower than those observed in non-irradiated bone (98.52%) [76]. The reported incidence of osteoradionecrosis is approximately 3% [77].

Augmented reality (AR) is also emerging as a valuable tool for preoperative planning. Before surgery, AR platforms can integrate multimodal imaging data, including computed tomography, magnetic resonance imaging, and endoscopic video, to generate three-dimensional anatomical reconstructions that surgeons can explore and manipulate within a virtual environment. This enables rehearsal of complex resections and simulation of reconstructive strategies before the patient enters the operating room [78]. When combined with dynamic navigation systems, AR may also be valuable in implant dentistry by allowing the surgeon to simultaneously visualize the surgical field and the virtual implant plan projected onto augmented reality glasses, thereby reducing visual distractions and minimizing overlay errors [79].

Other innovative approaches are also being investigated to improve oncologic treatment, including the therapeutic use of microRNAs (miRNAs). These small RNA molecules regulate the expression of numerous target genes, making them promising therapeutic candidates. Specific miRNAs are overexpressed in oral cancer and function as oncogenes, whereas others act as tumor suppressors. Oncogenic miRNAs represent therapeutic targets for reducing their activity and inhibiting molecular pathways involved in cancer progression. Conversely, restoring the expression of tumor-suppressor miRNAs may help counteract the aggressive biological behavior of oral cancer cells [80].

Chemotherapeutic applications of bioactive phenolic compounds also show considerable promise. Phenolic compounds can interfere with cancer progression at multiple stages, from uncontrolled cell proliferation to invasion and metastasis, making them attractive candidates as anticancer agents. In addition, phenolic compounds may enhance the efficacy of existing chemotherapeutic drugs and represent a potential strategy for overcoming chemoresistance [81].

### Limitations of this review

This review has several limitations that should be acknowledged. First, the available evidence is largely derived from case reports and small retrospective studies, which inherently provide lower levels of evidence and are more susceptible to bias. High-quality prospective studies and randomized controlled trials evaluating prosthetic rehabilitation in patients with oral cancer remain scarce, limiting the strength of the conclusions that can be drawn. In addition, the considerable heterogeneity in prosthetic designs, rehabilitation protocols, outcome measures, and follow-up periods reduces the comparability of published studies and restricts the generalizability of the findings.

As this was a narrative review, the selection of studies was based on the authors’ critical appraisal, which may have introduced selection bias. To minimize this limitation, all potentially eligible studies and any disagreements regarding study inclusion were discussed and resolved by consensus among the co-authors. Furthermore, although English-language publications comprise the majority of the available literature, the exclusion of studies published in other languages may have resulted in language bias.

Future research should prioritize well-designed multicenter prospective studies with standardized outcome measures and longer follow-up periods to provide more robust evidence regarding the long-term performance, survival, and clinical effectiveness of different prosthetic rehabilitation strategies for patients with oral cancer.

## Conclusion

Prosthetic rehabilitation plays a central role in the social reintegration of patients undergoing oncologic treatment, significantly improving quality of life by restoring masticatory, speech, and swallowing functions. Given the wide range of anatomical and functional alterations resulting from oncologic treatment, dentists must be well informed about the available prosthetic strategies to address these challenges. A multidisciplinary approach is essential to ensure a comprehensive, effective, and integrated treatment plan for these patients.

## Data Availability

No datasets were generated or analyzed during the current study.
